# Prognostic Factors and Survival Outcomes in Resected Biliary Tract Cancers: A Multicenter Retrospective Analysis

**DOI:** 10.3390/cancers17152445

**Published:** 2025-07-23

**Authors:** Michele Ghidini, Fausto Petrelli, Matteo Paccagnella, Massimiliano Salati, Francesca Bergamo, Margherita Ratti, Caterina Soldà, Barbara Galassi, Ornella Garrone, Massimo Rovatti, Arianna Zefelippo, Lucio Caccamo, Enrico Gringeri, Alessandro Zerbi, Guido Torzilli, Silvia Bozzarelli, Lorenza Rimassa, Gianluca Tomasello

**Affiliations:** 1 Medical Oncology Unit, Fondazione IRCCS Ca’ Granda Ospedale Maggiore Policlinico, 20122 Milan, Italy; barbara.galassi@policlinico.mi.it (B.G.); ornella.garrone@policlinico.mi.it (O.G.); 2 Oncology Unit, ASST Bergamo Ovest, 24047 Treviglio, Italy; faupe@libero.it; 3 A.O. Santa Croce e Carle, CTC, 12100 Cuneo, Italy; matteo.babeuf@gmail.com; 4 Department of Oncology and Hematology, University Hospital of Modena, 41124 Modena, Italy; maxsalati@live.it; 5 Medical Oncology 1 Unit, Veneto Institute of Oncology IOV–IRCCS, 35128 Padova, Italy; francesca.bergamo@iov.veneto.it (F.B.); caterina.solda@iov.veneto.it (C.S.); 6 Oncology Unit, ASST of Cremona, 26100 Cremona, Italy; m.ratti2@ausl.pc.it; 7 Surgery Unit, ASST of Cremona, 26100 Cremona, Italy; massimo.rovatti@asst-cremona.it; 8 Liver Transplant Unit, Fondazione IRCCS Ca’ Granda Ospedale Maggiore Policlinico, 20122 Milan, Italy; arianna.zefelippo@policlinico.mi.it (A.Z.); lucio.caccamo@policlinico.mi.it (L.C.); 9 Hepatobiliary Surgery and Liver Transplantation Unit, Department of Surgery, Oncology and Gastroenterology, University of Padova, 35128 Padova, Italy; enrico.gringeri@unipd.it; 10 Department of Biomedical Sciences, Humanitas University, 20072 Pieve Emanuele, Italy; alessandro.zerbi@hunimed.eu (A.Z.); guido.torzilli@hunimed.eu (G.T.); lorenza.rimassa@hunimed.eu (L.R.); 11 Pancreatic Unit, IRCCS Humanitas Research Hospital, 20089 Rozzano, Italy; 12 Department of Hepatobiliary and General Surgery, IRCCS Humanitas Research Hospital, 20089 Rozzano, Italy; 13 Medical Oncology and Hematology Unit, Humanitas Cancer Center, IRCCS Humanitas Research Hospital, 20089 Rozzano, Italy; silvia.bozzarelli@cancercenter.humanitas.it; 14 Oncology Unit, ASST Crema, 26013 Crema, Italy; gianluca.tomasello@gmail.com

**Keywords:** biliary tract cancers, adjuvant chemotherapy, intrahepatic cholangiocarcinoma, extrahepatic cholangiocarcinoma, gallbladder cancer

## Abstract

**Background/Objectives**: Biliary tract cancers (BTCs) are aggressive malignancies with a poor prognosis. Surgery remains the only curative option, yet recurrence rates are high, and the role of adjuvant chemotherapy remains debated. This study aims to evaluate the impact of adjuvant chemotherapy and prognostic factors on survival outcomes in resected BTCs. **Methods**: We conducted a retrospective multicenter study analyzing patients diagnosed with intrahepatic (iCCA) and extrahepatic cholangiocarcinoma (eCCA) or gallbladder cancer (GBC) who underwent curative-intent surgical resection between 1999 and 2023. Demographic, clinicopathological, and treatment data were collected from institutional databases. Survival outcomes were assessed using Kaplan–Meier analysis, and prognostic factors were identified through Cox proportional hazards regression. **Results**: A total of 155 patients were included, with a median follow-up of 84.6 months. The cohort comprised 38.7% iCCA, 31.6% eCCA, and 29.7% GBC. R0 resection was achieved in 77.4% of cases, while lymph node involvement was present in 39.4%. Median overall survival (OS) significantly varied by stage (*p* < 0.001), ranging from >60 months for stage I to ~12 months for stage IVA. Eastern Cooperative Oncology Group (ECOG) performance status (PS) emerged as the strongest independent prognostic factor for OS (*p* < 0.001). Adjuvant chemotherapy, administered to 49.0% of patients, did not significantly improve OS in the overall cohort (*p* = 0.899). However, subgroup analyses suggested potential benefits in iCCA and eCCA but not in GBC. High CA19-9 levels and vascular invasion were associated with poorer survival outcomes. **Conclusions**: This study highlights the prognostic significance of ECOG PS, resection margin status, lymph node involvement, and CA19-9 levels in resected BTCs. The lack of a clear survival benefit from adjuvant chemotherapy underscores the need for improved therapeutic strategies. Future research should focus on refining risk stratification models and identifying more effective adjuvant treatments to enhance long-term survival outcomes in patients with BTC.

## Simple Summary

The role of adjuvant chemotherapy in resected biliary tract cancers (BTCs) is a matter of debate. With our retrospective multicenter study, we evaluated the impact of adjuvant chemotherapy and prognostic factors on survival outcomes in resected BTCs. Eastern Cooperative Oncology Group (ECOG) performance status (PS) emerged as the most powerful predictor of OS, reinforcing the critical role of functional status in determining patient prognosis. Resection with clear margins (R0) remained a strong predictor of improved survival, highlighting the importance of surgical radicality. CA19-9 levels retained independent prognostic value, suggesting that this biomarker could be integrated into routine clinical practice for risk assessment. While tumor stage and lymph node status were also associated with survival, their impact was less pronounced in the multivariate model, possibly reflecting the complex interplay of multiple prognostic factors. Adjuvant chemotherapy, administered to 49.0% of patients, was not associated with OS improvement.

## 1. Introduction

Biliary tract cancers (BTCs), which include intrahepatic cholangiocarcinoma (iCCA), extrahepatic cholangiocarcinoma (eCCA) and gallbladder cancer (GBC), are rare but highly aggressive malignancies that arise from the epithelial lining of the bile duct. Although they account for a small percentage of all gastrointestinal cancers, their incidence appears to be increasing worldwide, particularly in the intrahepatic forms. These neoplasms are often characterized by non-specific symptoms and insidious onset, leading to delayed diagnosis and a high proportion of patients presenting with locally advanced or metastatic disease at the time of clinical evaluation. As a result, long-term survival rates remain poor, and the five-year overall survival (OS) rate is often less than 20% in unselected populations [1,2].

Surgical resection remains the only potentially curative treatment option for BTCs. However, even in patients who undergo curative surgery, the prognosis is often guarded due to high recurrence rates. The presence of unfavorable pathologic features (such as positive resection margins, lymph node involvement and vascular invasion) or biological factors, such as elevated preoperative CA19-9 levels, accounts for the dismal outcome. In addition, the biological heterogeneity of BTCs and their anatomical complexity pose a major challenge for both diagnosis and management. The role of adjuvant therapy in BTCs is the subject of ongoing debate. Although several randomized controlled trials and meta-analyses have attempted to define the benefits of adjuvant chemotherapy, the results have been inconclusive [3,4]. Current guidelines of the European Society of Medical Oncology recommend adjuvant treatment but rely on limited and heterogeneous data [5]. Apart from the phase III BILCAP and ASCOT trials, which showed an advantage in survival outcomes given by adjuvant chemotherapy [6,7,8], most studies include relatively small sample sizes or do not differentiate between BTC subtypes, leading to uncertainties regarding the optimal therapeutic approach for each group [6,7,8,9,10,11,12].

In this context, the identification of robust and clinically applicable prognostic factors is essential to guide postoperative management and improve risk stratification. Several variables have emerged as potential predictors of OS and recurrence-free survival (RFS), including nodal involvement and margin status [13,14,15,16,17]. However, the prognostic significance of each of these factors may vary depending on tumor location, histologic subtype and treatment setting. In addition, the decision to use adjuvant chemotherapy is often influenced by multiple patient- and tumor-related variables, making real-world data particularly valuable for understanding treatment patterns and outcomes outside of clinical trials.

This multicenter retrospective study aims to analyze a large cohort of patients with resected BTCs treated at multiple Italian Institutions over a period of two decades. This study has three objectives: first, to evaluate long-term survival rates after surgical resection; second, to identify independent prognostic factors associated with OS and RFS; and third, to evaluate the impact of adjuvant chemotherapy on survival in different BTC subtypes. By integrating clinical, pathologic and treatment-related data with long-term follow-up, this study provides a comprehensive overview of survival factors in a specific cohort of patients with resected BTCs. The results of this study may help to refine clinical decision making, improve patient selection for adjuvant therapies and ultimately provide insights useful to improve the quality of care for this challenging and diverse group of malignancies.

## 2. Materials and Methods

### 2.1. Study Design and Population

This retrospective multicenter study analyzed patients diagnosed with iCCA, eCCA and GBC who underwent surgical resection across 7 Italian Institutions. Data were collected from institutional medical records and oncological databases, including demographic information, tumor characteristics, treatment modalities, and survival outcomes. Patients diagnosed between 1999 and 2023 were included. Patients were eligible if they had histologically confirmed CCA or GBC and had undergone surgical resection with curative intent. Exclusion criteria included patients with distant metastases at diagnosis, those with incomplete medical records, or those who received only palliative care without surgery.

### 2.2. Data Collection

Patient data were extracted from electronic health records and institutional databases. The following demographics and tumor variables were collected: age, sex, comorbidities, ECOG PS, primary tumor location (iCCA, eCCA, GBC), stage at diagnosis according to the 8th edition of the American Joint Committee on Cancer (AJCC) TNM classification, vascular invasion evaluated on histological sample, resection margin status (R0, R1), tumor grading and lymph node dissection results. Biomarker assessment involved CA19-9 levels at diagnosis. Treatment modalities included surgical resection type, administration of adjuvant therapy and type of adjuvant chemotherapy (5-FU/capecitabine, gemcitabine-based regimens). Survival outcomes included OS, which was defined as the time from diagnosis to death or last follow-up, and RFS, which was defined as the time from the date of curative surgery to the time of recurrence.

The study cohort was divided into four stages: Stage I (localized, early-stage disease); Stage II (localized disease without lymph node involvement); Stage III (lymph node involvement or extensive local disease); and Stage IVA (locally advanced, unresectable disease without distant metastasis). The ECOG PS was used to evaluate patients’ baseline functional capacity and its impact on survival. Adjuvant chemotherapy was administered based on institutional protocols and at the physician’s discretion. Chemotherapy regimens included 5-FU/capecitabine-based or gemcitabine-based treatments. Patients were followed with periodic imaging and CA19-9 biomarker assessment based on the local practices of each participating institution. Median follow-up duration was 84.6 months.

### 2.3. Statistical Analysis

Descriptive statistics were used to summarize patient characteristics. Kaplan–Meier survival analysis was performed to estimate median OS and RFS. Log-rank tests were used to compare survival distributions across different subgroups (tumor stage, resection status, adjuvant chemotherapy). Cox proportional hazards regression analysis was conducted to identify independent prognostic factors for OS and RFS. Covariates included ECOG PS, tumor stage, resection margin status, lymph node involvement, vascular invasion, CA19-9 levels, and adjuvant chemotherapy. All statistical analyses were performed using SPSS version 24.0 (IBM, New York, NY, USA) or R software (version 4.4.0) (R foundation for statistical computing, Vienna, Austria). A *p*-value < 0.05 was considered statistically significant.

### 2.4. Ethical Considerations

This study was conducted in accordance with the Declaration of Helsinki. Ethical approval was obtained from the participating centers’ Institutional Review Board (IRB). Patient confidentiality was maintained, and informed consent was waived due to the study’s retrospective nature.

## 3. Results

The study included 155 patients diagnosed with CCA or GBC who underwent surgical resection. Patients’ characteristics are summarized in Table 1. The tumor types were categorized as follows: 38.7% iCCA, 31.6% eCCA, and 29.7% GBC. The median age was 66.4 years, ranging from 21 to 88 years. The gender distribution was nearly balanced, with 50.3% male and 49.7% female patients. ECOG PS 0 was reported in 49% of patients, while 33.5% scored 1 and 17.4% scored 2. At diagnosis, tumor staging revealed 14.8% as stage I, 32.3% as stage II, 47.1% as stage III, and 5.8% as stage IVA, with most patients presenting with advanced disease. Surgical outcomes indicated that 77.4% of patients underwent R0 resection (negative margins), while 22.6% had R1 resections (microscopic residual disease). Lymph node involvement was seen in 39.4% of patients (35.5% N1, 2.6% N2), and vascular invasion was present in 44.5% of cases (Table 1 and Table 2).

### 3.1. Prognostic Covariates

Regarding stage and nodal involvement, tumor staging significantly influenced survival outcomes: median survival was >60 months for stage I, moderately reduced for stage II, <24 months for stage III, and approximately 12 months for stage IVA. Resection status also significantly impacted survival, with markedly improved outcomes following R0 versus R1 resections, particularly in GBC (*p* < 0.001). Lymph node involvement was associated with significantly poorer survival outcomes (*p* = 0.027).

**Figure 1:**
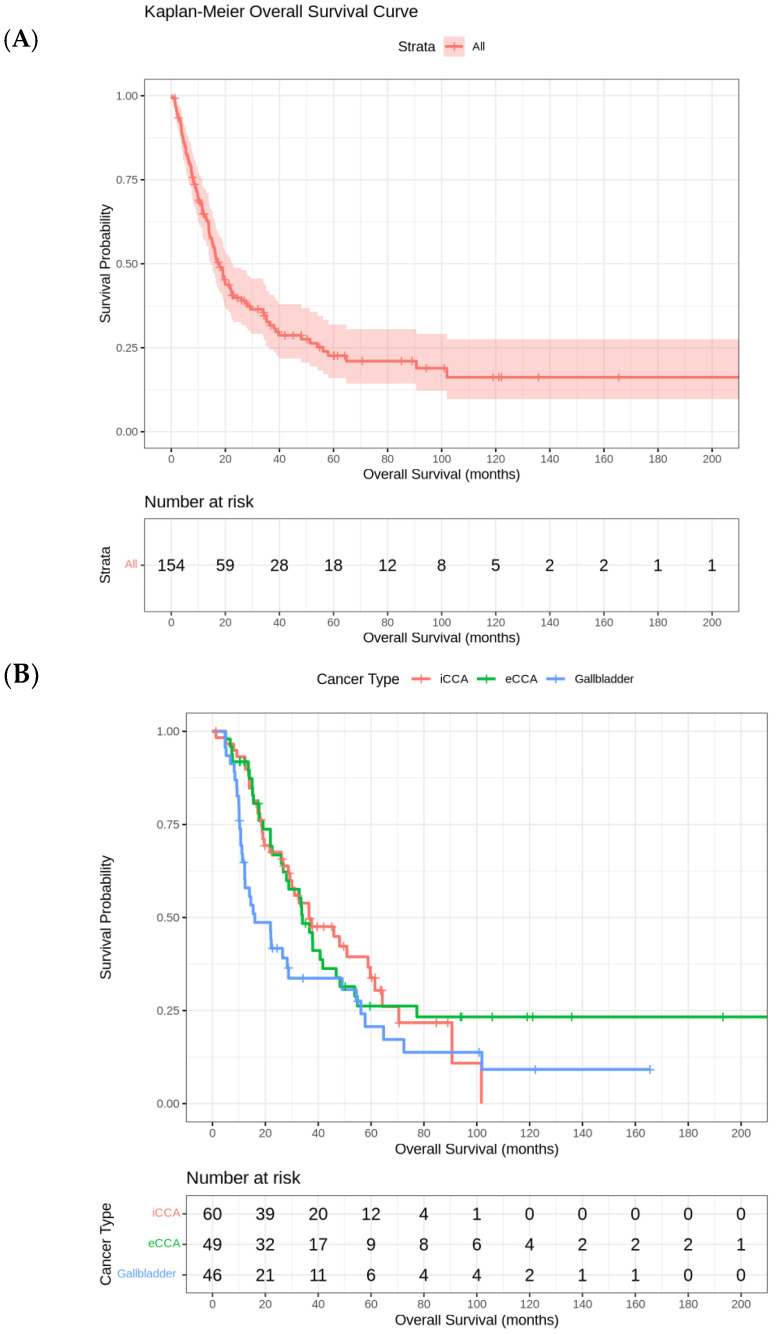
(**A**) Kaplan–Meier Curve for overall survival. (**B**) Kaplan–Meier curves for overall survival by tumor site (iCCA, eCCA, GBC).

Kaplan–Meier curve for OS and Kaplan–Meier curves for OS by tumor site (iCCA, eCCA, GBC) are reported in Figure 1A and Figure 1B, respectively.

ECOG PS strongly influenced OS: patients with an ECOG PS 0 had a median OS of approximately 61 months (95% CI: 49.1–101.7 months), with ECOG 1 of 22 months (95% CI: 17.97–28.83 months), and with ECOG 2 of 9 months (95% CI: 7.57–12.47 months) (Table 3 and Table 4).

Median survival by chemotherapy administration was 59.7 months (95% CI: 34.1–85.3) with chemotherapy and 29.3 months (95% CI: 18.6–40.0) without chemotherapy in iCCA; 40.7 months (95% CI: 25.5–55.9) with chemotherapy and 32.8 months (95% CI: 17.3–48.4) without chemotherapy in eCCA; 12.2 months (95% CI: 7.1–17.3) with chemotherapy versus 28.3 months (95% CI: 13.0–43.6) without chemotherapy in GBC. Adjuvant chemotherapy was administered to 76 patients (49.0%) and did not significantly improve OS (*p* = 0.899). Univariate Kaplan–Meyer analysis by BTCs subgroups indicated borderline non-significant survival benefits in favor of the observation group in iCCA (*p* = 0.0784) and in favor of chemotherapy in eCCA (*p* = 0.0781). No significant benefit was registered in GBC patients receiving adjuvant chemotherapy (*p* = 0.543) (Supplementary Figures S1 and S2).

Younger patients (<65 years) more frequently received adjuvant chemotherapy. Age-related survival outcomes varied; younger patients with eCCA had better median survival (48.2 months [95% CI 18.6–77.8] vs. 28.8 months [95% CI 19.6–38.1] in older patients). Conversely, older patients showed slightly better outcomes in iCCA (50.9 months [95% CI 21.4–80.4] vs. 32.8 months [95% CI 21.3–44.3]) and GBC (28.8 months [95% CI 0–58.2] vs. 10.7 months [9.1–12.3]).

Vascular invasion significantly negatively affected survival, particularly in iCCA (*p* = 0.0005).

Finally, to explore potential changes in outcomes over time, potentially introducing the risk of temporal bias, we performed an analysis of patients stratified into two cohorts according to enrollment period (i.e., 1999–2010 vs. 2011–2023). A significant survival difference was found in favor of patients treated more recently (n = 101 vs. 53). Specifically, median OS was 18 months (IQR 10.0–36.7) in patients treated from 2011 to 2023 as compared to 11.4 months (IQR 5.0–22.2) for patients treated from 1999 to 2010.

### 3.2. Multivariable Cox Regression Analysis

This analysis confirmed the independent prognostic significance of several key factors. ECOG PS emerged as the most powerful predictor of OS, reinforcing the critical role of functional status in determining patient prognosis. R0 resection remained a strong predictor of improved survival, highlighting the importance of surgical radicality. CA19-9 levels retained independent prognostic value, suggesting that this biomarker could be integrated into routine clinical practice for risk assessment. While tumor stage and lymph node status were also associated with survival, their impact was less pronounced in the multivariate model, possibly reflecting the complex interplay of multiple prognostic factors. Adjuvant chemotherapy administration was not associated with OS (Table 5).

### 3.3. RFS of the Entire Cohort

**Figure 2:**
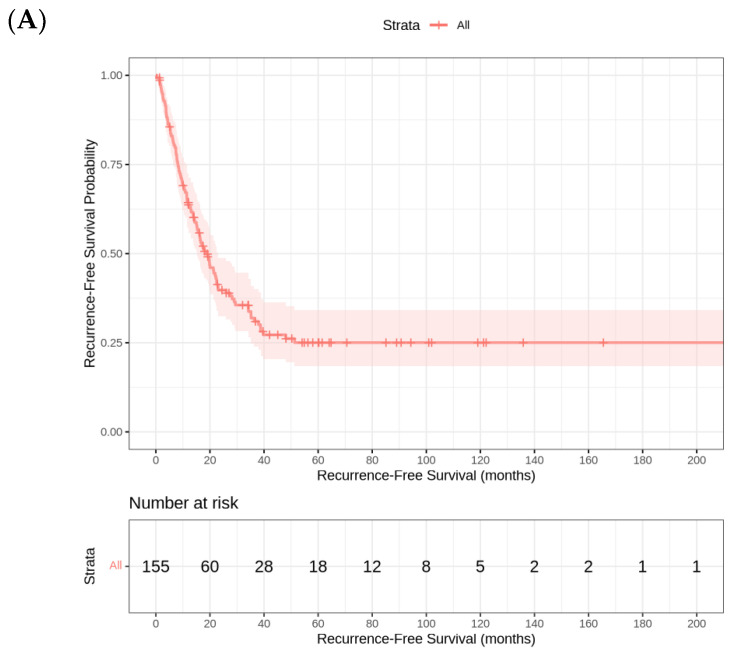

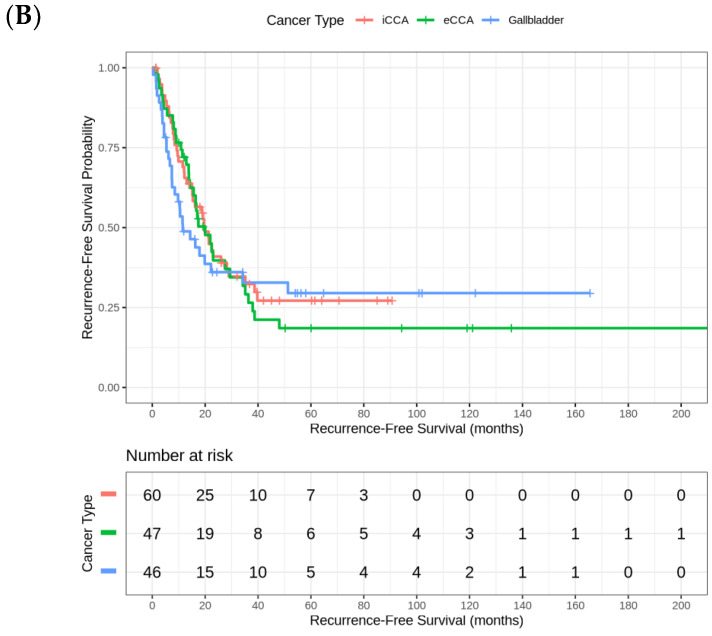
(**A**) Kaplan–Meier Curve for relapse-free survival. (**B**) Kaplan–Meier curves for relapse-free survival by tumor site (iCCA, eCCA, GBC).

The median RFS in this cohort is 18.73 (95% CI 14.3–22.5) months. The analysis of RFS by tumor site reveals significant differences across the three groups. Patients with eCCA iCCA had the longest median RFS (20 months [95% CI 13.2–26.8]), followed by iCCA (19.8 months [95% CI 13.9–25.7]). In contrast, patients with GBC experienced the shortest median RFS (11.5 months [95% CI 4.4–18.7]). The final Cox regression model revealed that ECOG PS was the strongest independent prognostic factor for RFS, with a hazard ratio (HR) of 2.83 (*p* < 0.001). This finding suggests that patients with worse functional status (higher ECOG scores) have a significantly increased risk of recurrence compared to those with better PS. The strong association between ECOG PS and RFS highlights the importance of the baseline patient condition in predicting long-term oncological outcomes (Figure 2).

## 4. Discussion

BTCs are highly aggressive malignancies that pose significant oncological challenges. Surgery remains the only curative option, but only a small proportion of patients are eligible for resection primarily due to their advanced stage at diagnosis. Long-term outcomes following potentially curative resection for BTC vary based on factors such as tumor location, stage, extent of surgery, comorbidities, and treatment-related complications. Current key prognostic factors include histologic margin status and lymph node involvement 1–3.

This retrospective multicenter study offers valuable insights into the prognostic factors and survival outcomes associated with a large cohort of patients with BTCs, highlighting the complex interplay of patient demographics, tumor characteristics, and treatment modalities.

In our cohort, tumor stage at diagnosis was a critical determinant of survival. As expected, patients diagnosed at earlier stages (i.e., stage I and II) exhibited significantly better outcomes compared to those with advanced disease (i.e., stage III and IVA). This finding underscores the importance of early detection and intervention, which remain challenging given the often asymptomatic nature of these cancers in their initial stages. The majority of patients presented with stage III disease, reflecting the aggressive biology of BTCs and the difficulty in achieving early diagnosis. The pronounced survival difference across stages emphasizes the need for improved diagnostic tools and strategies to identify these malignancies at a more treatable stage.

Surgical resection with negative margins (R0) was strongly associated with improved survival across all stages, reinforcing the central role of complete tumor removal in achieving curative outcomes. Conversely, microscopic residual disease (R1) was linked to significantly worse survival, particularly in GBC. For patients with early-stage disease, complete removal with negative macroscopic and microscopic margins remains the only potential curative strategy. However, the feasibility of surgical resection depends on tumor location, disease extent, patient comorbidities and individual surgeon experience, underscoring the importance of multidisciplinary evaluation. Therefore, early diagnosis and prompt referral to specialized high-volume centers are crucial to ensure optimal surgical outcomes and access to comprehensive care pathways [18]. This also suggests a potential role for neoadjuvant therapies in cases where complete resection is clearly not feasible. Indeed, while the use of neoadjuvant treatment has been increasing in other common cancers, its use in BTCs remains relatively rare [19,20,21,22]. Thus, there is a sound rationale for the use of a neoadjuvant systemic approach. As previously detailed, only a minority of patients presenting with BTCs are eligible for resection; fewer patients successfully undergo an R0 resection and the ability to achieve margin-negative resection is a primary determinant of long-term survival outcomes. Therefore, a greater impetus for pursuing preoperative treatment strategies with the potential to downstage locally advanced cancers and convert to resectable disease is warranted. In this direction, the phase II/III randomized trial PURITY is currently investigating a combination chemotherapy with cisplatin, gemcitabine and nab-paclitaxel in the preoperative setting for patients with resectable BTCs at high risk for recurrence. Control arm is standard surgery, primary endpoint is 12-month PFS for phase II part and PFS for the phase III study [23]. Moreover, next-generation sequencing of patients with advanced and refractory tumors has led to an improved understanding of the genetic changes driving cholangiocarcinogenesis and to the identification of key biomarkers for highly effective targeted therapies [24,25]. Clearly, more data are needed, preferably through well-designed prospective clinical trials incorporating novel agents, in order to define the indications for neoadjuvant therapy among patients with localized cholangiocarcinoma.

Lymph node involvement further worsened survival outcomes, with patients with negative nodes demonstrating markedly better outcomes than those with nodal metastases. These results highlight the necessity of thorough lymph node evaluation and dissection during surgery to ensure accurate staging and optimal treatment planning.

The ECOG PS emerged as a powerful independent prognostic factor for both OS and RFS. Patients with better functional status (ECOG PS 0) had significantly improved outcomes compared to those with higher ECOG PS scores, reinforcing the importance of baseline patient clinical conditions in predicting treatment response and long-term survival. This finding suggests that functional status should be a key consideration in treatment decision making and patient counseling, particularly in the context of aggressive malignancies where treatment tolerance and recovery are critical.

Elevated CA19-9 levels were strongly correlated with worse survival outcomes, particularly in iCCA. Consistent with previous reports [6], patients with increased CA19-9 levels before surgery had decreased long-term survival, with an impact comparable to node positivity and positive-margin resection. This reinforces the role of CA19-9 as a valuable prognostic biomarker, not only for initial risk stratification but also for monitoring disease relapse. It also suggests a potential role for its inclusion in current staging systems. Integrating CA19-9 with other molecular markers could further refine risk assessment and guide personalized treatment strategies, although this requires validation in larger, prospective studies.

The role of adjuvant chemotherapy in improving survival outcomes remains nuanced. While no significant OS benefit was observed in the entire cohort, subgroup analyses suggested potential benefits in specific tumor types, particularly eCCA. However, the lack of a clear benefit in iCCA and GBC raises questions about the efficacy of current systemic therapies for these subsets and highlights the need for more effective treatment strategies.

Age-related survival patterns varied by tumor type, with younger patients exhibiting better outcomes in eCCA, while older patients had a slight survival advantage in iCCA and GBC. This paradoxical finding may reflect differences in tumor biology, comorbidities, and treatment responses across age groups, warranting further investigation. Vascular invasion was a significant negative prognostic factor, particularly in iCCA, emphasizing the need for comprehensive preoperative imaging and possibly more aggressive treatment strategies in patients with vascular involvement.

Multivariate Cox regression analysis confirmed the independent prognostic significance of ECOG PS, resection margin status, and CA19-9 levels, while tumor stage and lymph node status had a less pronounced impact. The median RFS of 18.73 months for the entire cohort, with 103 recorded relapse events and significant differences across tumor sites, further highlights the aggressive nature of these malignancies. The strong association between ECOG PS and RFS underscores the importance of baseline patient condition in predicting long-term oncological outcomes.

As a retrospective study, our research is subject to potential biases, such as selection bias and information bias stemming from electronic data collection methods, as well as confounding factors, such as patient comorbidities and socioeconomic status. A further limitation is the small size of certain sample subgroups, which could reduce the statistical power of our analyses and limit the validity of conclusions drawn for these specific groups. Moreover, adjuvant chemotherapy was not administered using standardized criteria across centers, and the decision was influenced by physician judgment and institutional practices. This likely introduced selection bias, particularly favoring treatment for patients with more advanced stages, R1 resection, or younger age, as shown in Table 2.

These factors might affect the generalizability of our findings and the reliability of certain statistical analyses. However, we believe that our study provides valuable insights into a population of patients with BTCs treated at different hospital centers in Italy, contributing to meaningful comparisons with existing evidence given by the BILCAP study, with oral adjuvant capecitabine recommended in radically resected BTC [9,11]. Larger multicenter studies to validate these subgroup analyses and strengthen the obtained results are warranted.

## 5. Conclusions

In conclusion, this study underscores the multifactorial nature of survival in cholangiocarcinoma and gallbladder cancer, with tumor stage, resection status, lymph node involvement, PS, and CA19-9 levels playing pivotal roles. The results emphasize the need for early diagnosis, complete surgical resection, and personalized treatment strategies tailored to individual patient and tumor characteristics. Bile duct tumors do represent a difficult clinical challenge that requires specialized centers for proper management, preferably in the context of dedicated multidisciplinary teams in order to optimize the knowledge of each specialist in each field and to always offer patients the best treatment options, including innovative clinical trials [26,27]. Future research should focus on refining risk stratification models, possibly integrating genomic and molecular data, exploring novel therapeutic approaches, and validating these findings in larger, prospective cohorts to improve outcomes for patients with these challenging malignancies.

## Figures and Tables

**Table 1:** Patients’ characteristics.

Characteristic	Distribution (n, %)
**Age, mean (range)**	66.4 (21.0–88.0)
**Sex**	
Female	77 (49.7%)
Male	78 (50.3%)
**Tumor Type**	
Intrahepatic	60 (38.7%)
Extrahepatic	49 (31.6%)
Gallbladder	46 (29.7%)
**ECOG Performance Status**	
0	76 (49.0%)
1	52 (33.5%)
2	27 (17.5%)
**Resection Status**	
R0	120 (77.4%)
R1	35 (22.6%)
**T Stage**	
T0	1 (0.6%)
T1	22 (14.2%)
T1a	1 (0.6%)
T1b	3 (1.9%)
T2	58 (37.4%)
T2a	4 (2.6%)
T2b	8 (5.2%)
T3	48 (31.0%)
T4	10 (6.5%)
**N Stage**	
N0	94 (60.6%)
N1	55 (35.5%)
N2	4 (3.9%)
NX	2 (1.3%)
**V Status**	
V0	86 (55.5%)
V1	69 (44.5%)
**TNM Stage**	
I	23 (14.8%)
II	50 (32.3%)
III	73 (47.1%)
IVA	9 (5.8%)

**Table 2:** Patients’ characteristics according to no adjuvant/adjuvant treatment.

Characteristic	No Adjuvant (n = 79)	Adjuvant (n = 76)	*p* Value
**Age**	68.8 (41–88)	63.8 (21–86)	0.003
**Sex**			0.811
Female	38 (48.1%)	39 (51.3%)	
Male	41 (51.9%)	37 (48.7%)	
**Tumor Type**			0.301
Intrahepatic	31 (39.2%)	29 (38.2%)	
Extrahepatic	21 (26.6%)	28 (36.8%)	
Gallbladder	27 (34.2%)	19 (25.0%)	
**ECOG PS**			0.846
0	37 (46.8%)	39 (51.3%)	
1	28 (35.5%)	24 (31.6%)	
2	14 (17.7%)	13 (17.1%)	
**Resection Status**			0.012
R0	68 (86.1%)	52 (68.4%)	
R1	11 (13.9%)	24 (31.6%)	
**T Stage**			0.005 (T1 gropued, T2 grouped and T3/T4 grouped
T1	18 (22.8%)	4 (5.3%)	
T1a	1 (1.3%)	0 (0%)	
T1b	2 (2.5%)	1 (1.3%)	
T2	34 (43.0%)	24 (31.6%)	
T2b	1 (1.3%)	7 (9.2%)	
T3	21 (26.6%)	27 (35.5%)	
T4	2 (2.5%)	13 (17.1%)	
**N Stage**			0.018
N0	57 (72.2%)	37 (48.7%)	
N1	22 (27.8%)	33 (43.4%)	
N2	0 (0%)	4 (5.3%)	
NX	0 (0%)	2 (2.6%)	
**V Status**			0.053
V0	51 (64.6%)	35 (46.1%)	
V1	28 (35.4%)	41 (53.9%)	
**TNM Stage**			0.009
I	19 (24.1%)	4 (5.3%)	
II	31 (39.2%)	19 (25.0%)	
III	27 (34.2%)	46 (60.5%)	
IVA	2 (2.5%)	7 (9.2%)	

**Table 3:** Univariate log-rank test for OS.

Variable	Median OS (Months)	95% C.I.	*p*-Value
**Stage**			0.002
I	101.93	nr	
II	40.73	24.53–56.93	
III	19.13	9.73–28.54	
IV	17.97	9.30–26.64	
**Resection**			0.001
R0	36.70	28.06–45.34	
R1	13.97	12.08–15.86	
**N**			0.062
N0	37.93	25.06–50.80	
N1	15.53	12.17–18.90	
**ECOG-PS**			0.001
0	61.47	49.07–101.73	
1	21.93	17.97–28.83	
2	9.23	7.57–12.47	
**Adjuvant Chemotherapy**			0.899
yes	28.67	23.29–30.04	
no	33.77	21.55–45.98	
**Age**			0.952
≤65 years old	28.67	17.24–40.09	
>65 years old	33.77	25.00–42.53	
**V**			0.0027
yes	21.93	13.81–30.06	
no	48.23	33.52–62.94	

**Table 4:** Univariate Cox regression analysis for OS.

Variable	HR	95% C.I.	*p*-Value
**Ca19.9** (≤29 U/mL vs. >29 U/mL)	0.590	0.40–0.87	0.0071
**Resection** (R1 vs. R0)	2.05	1.35–3.12	0.0008
**Lymph Node Status** (N+ vs. N0)	1.53	1.04–2.26	0.0072
**Vascular Invasion** (V1 vs. V0)	1.8	1.23–2.63	0.0025
**ECOG PS** (1+ vs. 0)	4.91	3.22–7.48	<0.0001
**Adjuvant chemotherapy** (Yes vs. no)	1.026	0.70–1.50	0.897

**Table 5:** Multivariable Cox regression analysis for OS.

Variable	HR	95% C.I.	*p*-Value
**Ca19.9** (>29 U/mL vs. ≤29 U/mL)	1.36	0.92–2.03	0.127
**Resection** (R1 vs. R0)	2.11	1.34–3.33	0.001
**Lymph Node Status** (N0 vs. N+)	0.57	0.38–0.86	0.007
**Vascular Invasion** (V1 vs. V0)	1.70	1.14–2.54	0.010
**ECOG PS** (1+ vs. 0)	5.16	3.33–7.99	<0.0001
**Adjuvant chemotherapy** (Yes vs. no)	0.76	0.50–1.15	0.199

## Data Availability

The data presented in this study are available in this article and Supplementary Materials.

## Supplementary Materials

The following supporting information can be downloaded at: https://www.mdpi.com/article/10.3390/cancers17152445/s1, Figure S1: Kaplan-Meier Curve for overall survival by tumor type (no adjuvant treatment); Figure S2: Kaplan-Meier Curve for overall survival by tumor type (adjuvant treatment).

## Abbreviations

The following abbreviations are used in this manuscript:

BTCs	Biliary tract cancers
ECOG PS	Eastern Cooperative Oncology Group performance status
eCCA	Extrahepatic cholangiocarcinoma
ICCA	Intrahepatic cholangiocarcinoma
GB	Gallbladder carcinoma
