# Supplementary material for: Prognostic Factors and Survival Outcomes in Resected Biliary Tract Cancers: A Multicenter Retrospective Analysis
Source: Cancers (Basel). 2025 Jul 23;17(15):2445. doi: 10.3390/cancers17152445 (PMC12346334; doi:10.3390/cancers17152445)
Supplement: Supplementary file 1 [file cancers-17-02445-s001.zip › cancers-3730761-supplementary.pdf]

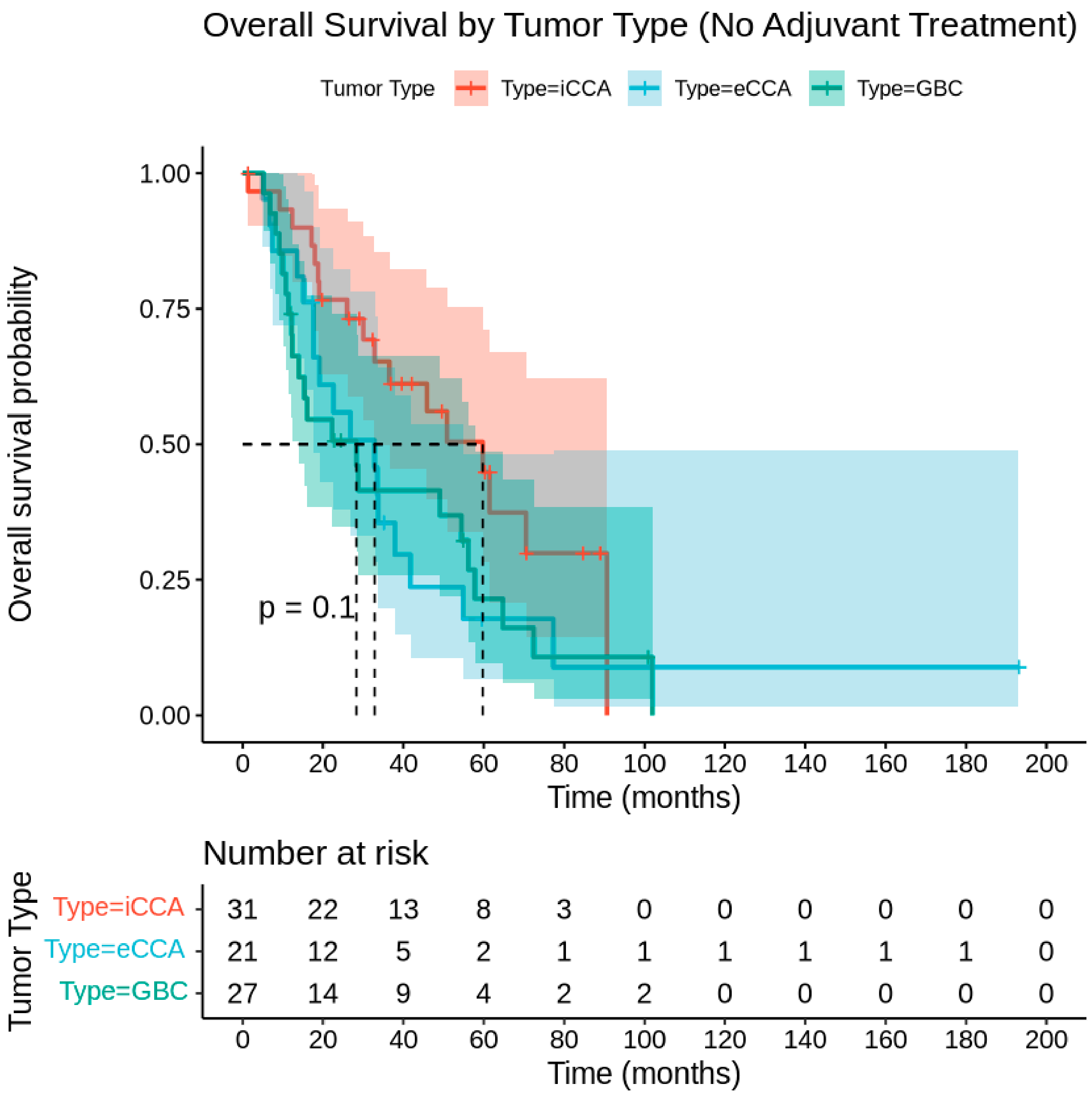

Supplementary Figure S1. Kaplan-Meier Curve for overall survival by tumor type (no adjuvant treatment).

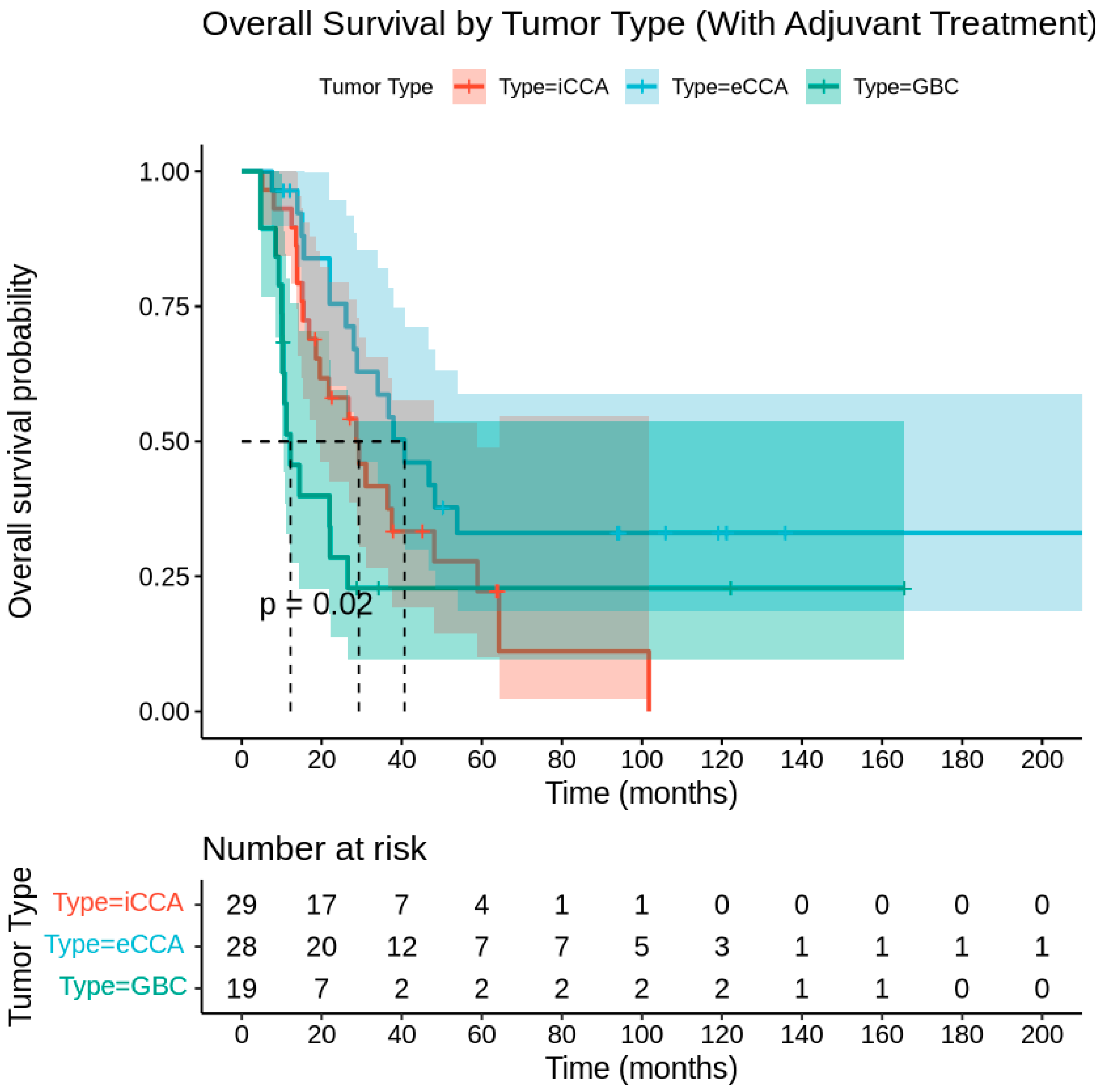

Supplementary Figure S2. Kaplan-Meier Curve for overall survival by tumor type (adjuvant treatment).
